# Detecting Intra-Fraction Motion in Patients Undergoing Radiation Treatment Using a Low-Cost Wireless Accelerometer

**DOI:** 10.3390/s90906715

**Published:** 2009-08-27

**Authors:** Farid Farahmand, Kevin O. Khadivi, Joel J. P. C. Rodrigues

**Affiliations:** 1 Department of Engineering Science, Sonoma State University, Rohnert Park, CA 94928, USA; E-Mail: farid.farahmand@sonoma.edu; 2 Austin Cancer Center, 2600 E. Martin Luther King Jr. Blvd., Austin, Texas 78702, USA;E-Mail: bepish@flash.net; 3 Instituto de Telecomunicações, Department of Informatics, University of Beira Interior, Covilhã, 6201-001, Portugal

**Keywords:** sensor networks, accelerometer, e-health, medical applications, medical systems

## Abstract

The utility of a novel, high-precision, non-intrusive, wireless, accelerometer-based patient orientation monitoring system (APOMS) in determining orientation change in patients undergoing radiation treatment is reported here. Using this system a small wireless accelerometer sensor is placed on a patient’s skin, broadcasting its orientation to the receiving station connected to a PC in the control area. A threshold-based algorithm is developed to identify the exact amount of the patient’s head orientation change. Through real-time measurements, an audible alarm can alert the radiation therapist if the user-defined orientation threshold is violated. Our results indicate that, in spite of its low-cost and simplicity, the APOMS is highly sensitive and offers accurate measurements. Furthermore, the APOMS is patient friendly, vendor neutral, and requires minimal user training. The versatile architecture of the APOMS makes it potentially suitable for variety of applications, including study of correlation between external and internal markers during Image-Guided Radiation Therapy (IGRT), with no major changes in hardware setup or algorithm.

## 1. Introduction

Advances in wireless sensor networks (WSNs) have created many new opportunities in healthcare and medical systems. Examples of WSN applications in such areas are monitoring of blood pressure and oxygenation, breathing, heart rate, heart rhythm, electroencephalograms (EEGs) and electrocardiograms (ECGs). Wireless sensor networks have also been considered for high-precision, non-intrusive position monitoring.

Patient position monitoring is particularly essential for accurately delivered radiation therapy treatments. With the advent of Stereotactic Radiation Therapy (SRT) and ever decreasing target margins, even a nearly imperceptible movement may have a substantial negative impact on a patient’s outcome. Vigilant active observation of patient movement serves two primary purposes: (1) it ensures that a patient who inadvertently moves during treatment will not receive an improperly directed dose; (2) it ensures that radiation is properly delivered, in spite of involuntary patient movements, e.g., breathing. Advanced Image Guided Radiation Therapy (IGRT) techniques, such as Dynamic Targeting, often utilize these cyclical patient movements to theoretically improve treatment accuracy [1]. Intrusive and/or cost-prohibitive methods are becoming less appealing as pain management throughout the treatment process gains more ground.

**Figure 1.**
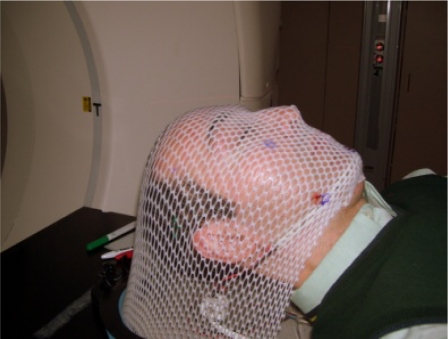
A typical setup to avoid head orientation change during radiotherapy using aquaplast thermoplastic (opti-mold) to provide pressure over the skin to immobilize head rotation.

Although considerable efforts are often undertaken to properly immobilize a patient, in many situations complete immobilization is not used or not possible. Figure 1 depicts a typical setup to immobilize patient’s head rotation for the entire duration of the radiotherapy, which is highly uncomfortable for the patient. Even when stringent immobilization precautions are taken, a patient’s head movements caused by physiological behaviours is generally extremely difficult, if not impossible, to prevent [2,3]. Similar studies have demonstrated that the problem is even more pronounced when patients are not thoroughly immobilized [4]. Despite such difficulties, few accurate head position monitoring systems currently exist. A passive visual monitoring by radiation therapists, using video cameras in the treatment room, is the prevalent conventional method. More advanced options are currently available, yet these methods are generally expensive and cumbersome, with a somewhat limited function.

In this preliminary study, we report our efforts in developing a wireless accelerometer-based patient orientation monitoring system (APOMS). The developed system is low-cost, low-power, small, and reusable and it has a highly flexible software platform. The main focus of this system is to accurately detect head orientation changes due to involuntary patient’s movement. The APOMS can potentially overcome many of the drawbacks associated with existing movement monitoring systems, such as setup complexity, high cost of maintenance and operation, or uncomfortable immobilization of the patient’s head as reported in [5]. Instead of imaging and calculating the movement of a small fiducial positioned on a patient, as is commonly utilized [2], an APOMS can be directly attached to the patient’s head to report change in orientation without any imaging by ionizing radiation. The accelerometer sensor in the APOMS has the capability to detect sub-degree rotation in all three orthogonal directions. The number of sensors can easily be expended in order to simultaneously monitor undesired sudden orientation changes of multiple body parts. Our test results indicate that in addition to being highly accurate and sensitive, the APOMS can perform well under high energy radiation conditions. Furthermore, while the APOMS provides sufficient signal strength for wireless communications, it does not interfere with radiation delivery.

As a final note, it must be emphasized that an APOMS is not capable of detecting and measuring any gradual linear movement. Thus, it cannot determine the actual amount of movement in the XYZ directions. Our proposed system is limited to measuring orientation and changes in orientation and detecting *sudden* positional changes. A more sophisticated body *positioning* system should be used to accurately measure movements in all six coordinates. In this experiment, however, we are only concerned with head orientation changes, since the body is typically secured during radiotherapy.

The remainder of this paper is structured as follow. In Section 2, we describe a typical clinical treatment environment. In Section 3, we elaborate on hardware description. In Section 4, we present details of the signal processing algorithm. In Section 5, we provide a general overview of the software implementation. Finally, in Section 6, we characterize the performance of APOMS, followed by concluding remarks.

## 2. Clinical Treatment Environment

**Figure 2.**
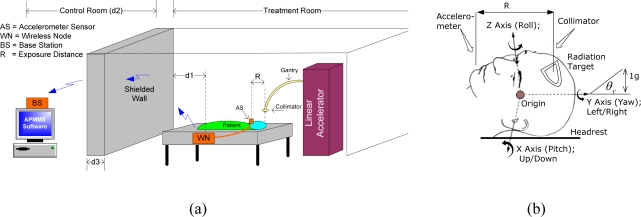
(a) Radiation treatment setup using the linear accelerator. (b) Head coordinates.

In this section we briefly describe the general setup used in a typical X-ray or radiation oncology (cancer therapy) procedure. Figure 2(a) depicts a typical brain cancer therapy treatment. The collimator, mounted on top of the gantry in the linear accelerator (linac), delivers radiation in a pre-determined pattern to the target. The APOMS consists of two parts: the wireless node (WN) and the base station (BS). The accelerometer sensor of the wireless node is attached to the patient in the treatment room, as shown in Figure 2(a). The base station is placed in the control room for monitoring the patient’s head orientation. As shown in Figure 2(a), the control room and the treatment room are completely isolated with a thick shielded wall. The shielding structure has a very high attenuation factor and it is designed to prevent personnel access and limiting exposure to scattered radiation from the collimator. The total distance between the RF antenna of the WN and the BS is equivalent to *d_1_ + d_2_ + d_3_*.

Figure 2(b) shows biodynamic coordinate system for human head (ISO-8727 defines the biodynamic coordinate system for human head. In this paper we reference roll, pitch, and yaw to Z, X, and Y coordinates, respectively, which are base on accelerometers’ coordinates). In this figure, one *g* displacement (90 degree rotation) is equivalent to the gravitational attraction that the Earth exerts on objects (e.g., the head) or near its surface, and it is approximately 9.80665 m/s^2^ or 32.1740 ft/s^2^. As shown in the figure, the accelerometer sensor and the radiation target are separated by distance R. We call this the *exposure distance*. Location of the sensor and selection of R value must be such that any change in orientation of the radiation target (patient’s head) can be accurately monitored and measured. In this figure, we assume the accelerometer sensor of the WN is attached to the chin of the patient.

## 3. Hardware Description

**Figure 3.**
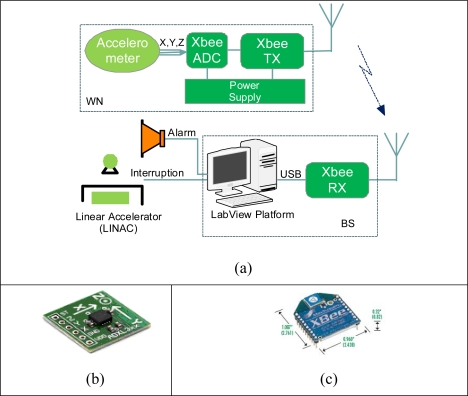
(a) Basic block diagram of APOMS; (b) ADXL330 accelerometer mounted on the breakout board; (c) Xbee transceiver.

Figure 3 depicts the main hardware components of APOMS prototype. The small (4 × 4 × 1.45 mm) accelerometer, mounted on a breadboard (2 × 2 cm), is attached to the patient’s body. The wireless communication between the accelerometer and the base station is supported by an Xbee transceiver [6]. Figure 3(a) shows the two main components of APOMS: WN and BS. In the following paragraphs we describe the functionality of each component in details.

### 3.1. Wireless Node (WN)

The wireless node (WN) consists of two separate parts wired together: the accelerometer sensor and the Xbee transceiver. The sensor, shown in Figure 3(b), is a small, low-g, low-power iMEMS accelerometer from Analog Devices (ADXL330) [7]. An accelerometer can measure both dynamic and static change of acceleration. The device is a polysilicon surface micromachined structure built on top of a silicon wafer [8]. When the structure moves, the dynamic changes of acceleration in all directions are detected using independent X, Y, and Z axes. Each axis reports the recent magnitude of acceleration using an analog voltage. The voltage is converted to an equivalent g value, which can be used to calculate the tilt angle. Consequently, the choice of accelerometer was based on its ability to achieve high degree resolution of tilt measurement and sensitivity within a measurement range of (+/−) 3g.

The outputs of the accelerometer are directed to three of 10-bit analog-to-digital converter (ADC) units on the XBee module, shown in Figure 3(c). The corresponding digital data is sent to the OEM RF module providing wireless data communication over ZigBee/802.15.4 protocol [9]. Hence, XBee modules operate in the license-free 2.4 GHz ISM band with a RF data rate of 250 kbps.

With the receiver sensitivity of −97 dBm, the typical transmission range of an Xbee module can be adjusted for 30–120 meters. Using XBee 802.15.4 the transmit power output can be boosted as high as 60 mW (+18 dBm). Each XBee module comes in either a regular or long-range “–PRO” version. In our design we used the PRO version to ensure maximum penetration through the shielded wall.

The Xbee module can be configured using a series of AT commands. More advanced features, such as supporting multi-node topology, data transmission, data reception, etc, can be configured using APIs (application programming interface). The configuration can be performed locally or over the air. In our design we implemented point-to-point configuration between WN and BS. The wireless node is powered by two AA batteries at about 3.3 V. For the best performance, it is important to use fully charged batteries.

### 3.2. Base Station (BS)

The base station consists of an Xbee transceiver connected to a PC. The Xbee transceiver directs the received digital data from the wireless node and passes it onto the PC via a USB interface. Using our own developed software, the received data is analyzed and displayed. As shown in Figure 3(a), the BS can be interfaced to the linear accelerometer and an audible alarm can notify technicians of any undesired patient change of orientation.

An important consideration in configuring the BS is properly setting its sensitivity level. The receiver sensitivity of XBee can be set to as low as −100 dBm. The following expression shows the relation between the receiver sensitivity, Φ_dBm_, in dBm and the transmitting power of the WN:

(1) $$\Phi_{\mathrm{dBm}}=10\text{log}\left[P_{\mathrm{tx}}\cdot [\alpha_{o}\left(d_{1}+d_{2}\right)+\alpha_{\mathrm{sh}}\left(d_{3}\right)\right],$$

where:

(2) $$\alpha_{o}\left(d_{1}+d_{2}\right)={\left[4\pi \cdot \left(d_{1}+d_{2}\right)\cdot f/C\right]}^{2}.$$

In the above expressions P*_tx_* is WN’s transmitted power in mW, *α*_sh_ is the attenuation factor of the shielded wall as a function of its thickness (d*_3_*) defined in [10], and *α_o_* is the free-space attenuation with d*_1_* and d*_2_* being the distance between the WN and BS, shown in Figure 2(a). In Equation (2)*, C* is the speed of light in free-space and *f* represents the license-free 2.4 GHz ISM band.

## 4. Signal Processing Algorithm

The received data from the accelerometer must be properly filtered and interpreted in order to accurately measure patient’s orientation. The signal processing algorithm constantly samples the accelerometer for static tilt information.

**Figure 4.**
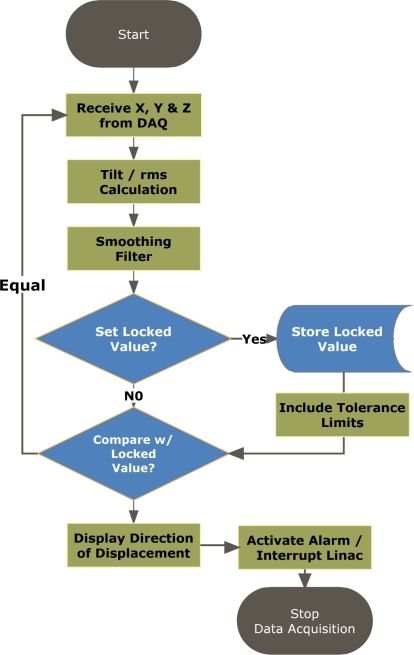
Flowchart of the signal processing algorithm used in APOMS.

Figure 4 depicts the flowchart for the signal processing algorithm. Initially, the received data from the accelerometer must be converted to *tilt angle* in order to properly determine the head rotation around X, Y, Z axes. Hence, the measured values from the accelerometer is compared to the zero *g* offset to determine if it is a *positive* or *negative* acceleration, e.g., if value is greater than the *offset* then the acceleration will indicate a positive acceleration, so the offset is subtracted from the value and the resulting value is passed to a tilt equation to determine the corresponding degree of tilt.

The following expressions are used to determine the tilt angle:

(3) $$\theta_{\text{deg}}={\text{sin}}^{-1}\frac{\left(V_{\mathrm{measured}}-V_{\mathrm{offset}}\right)}{\varphi };$$

or:

(4) $$\theta_{\mathrm{rad}}=\frac{\pi }{180}{\text{sin}}^{-1}\frac{\left(V_{\mathrm{measured}}-V_{\mathrm{offset}}\right)}{\varphi };$$

In the above expressions V*_measured_* is the accelerometer readout sampled by the ADC channel in mV. Furthermore, φ represents accelerometer’s sensitivity in mV/g. The value of V*_offset_* indicates the accelerometer’s zero *g* offset. In our application, value of V*_offset_* for each axis was found independently. Note that the sign of *θ* determines the direction of the tilt (e.g., up/down or left/right). Using Equation (3), when the tilt angle is zero degrees (zero *g*), V*_measured_* = V*_offset_*. The sensitivity value can be obtained from taking the difference between the measured values from the accelerometer at zero and 90 degrees, corresponding to zero and one g. Hence, the sensitivity can be calculated as follows:

(5) $$\varphi =\frac{\Delta V}{\Delta g}=V_{\mathrm{measured}}\;(g=1)-V_{\mathrm{measuredt}}\;(g=0).$$

Using the above expressions, with a measured sensitivity value of 270 mV/g and having 10-bit ADC embedded in the Xbee transceiver powered by a 3.3 V power supply, it can be demonstrated that APOMS can accurately offer 0.23 degree resolution at zero degree. Lower resolution is expected as the value of g increases.

As an example, consider the case where patient’s head rotates along X-axis (tilting up or down), as shown in Figure 2(b). For X-axis, the sensitivity, *φ*, has been measured to be 800 mV/g and V*_offset_* (g = 0) is determined to be 1,650 mV. After an upward head tilt, assuming the accelerometer reads 1,750 mV, we can calculate the tilt angle of the head around X-axes to be about 7.2 degrees. This indicates that the patient’s head rotated 7.2 degrees upwards compared to its initial coordinates.

The received raw data from WN is sampled at a rate of 200 Hz. This sampling rate is sufficient to detect any change in orientation of the patient’s head. However, the accelerometer is susceptible to noise. In order to ensure filtering the erroneous values measured by the accelerometer, the signal processing algorithm computes a running average to record the mean measured raw data over a period of one second. The choice of one second time interval was tested to be a good compromise between accurately obtaining data, while quickly detecting any changes in orientation. The latest average tilt value is calculated using the following expression:

(6) $${\bar{\theta }}_{s}=\frac{\sum_{i=s}^{1}\left(\theta_{i}-\theta_{i-1}\right)}{s},$$

where *s* is the data sampling rate (200 samples per second). In the above equation, *θ̄_s_* and *θ̄*_1_ represent the latest and the first recorded (displayed) tilt angle, respectively, in the running average window. The running root-mean-square (rms) average of *θ̄_s_* for all three axes is also computed using:

(7) $${\bar{\theta }}_{\mathrm{rms}}\;(\mathrm{xyz})=\sqrt{\bar{\theta }{\left(x\right)}^{2}+\bar{\theta }{\left(y\right)}^{2}+\bar{\theta }{\left(z\right)}^{2}}.$$

As shown in Figure 4, each average tilt value, including the rms average, is compared with the previous value to detect any change in orientation. Additional tolerance limits, *θ_th_*, can be assigned to each axis for allowing a small acceptable tilt. Consequently, having *θ̄_s_* > *θ̄_s_*_−1_+*θ_th_* indicates the patient rotating in the positive direction, where as having *θ̄_s_* < *θ̄_s_*_−1_−*θ_th_*, represents rotation in the negative direction. Under either condition, the interrupt circuitry and/or the alarm can be activated.

## 5. Software Implementation

Various software interfaces can be implemented to communicate with the Xbee module and process the signals from the accelerometer. In our prototype, we used LabVIEW programming language [11] for its ease of programming and versatility in data acquisition. LabVIEW is a graphical program development application developed by National Instruments. LabVIEW program consists of two parts: the control panel, providing the graphical user interface (GUI) and the block diagram, containing all the programming codes.

**Figure 5.**
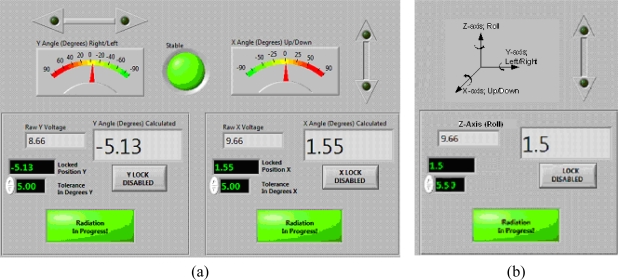
Control panel of APOMS using LabVIEW platform monitoring rotations about (a) X and Y axes and (b) Z axis.

Figure 5 shows the control panel designed for the APOMS. In this figure we only depict X- and Y-axis monitors due to space limitation.

Upon pressing the *Lock* button on the control panel, the user enables a reference orientation about X, Y, Z axes (e.g., *θ°*(*x*)) desired for radiation. For each independent axis a (+/−) tolerance limit can be assigned, allowing the patient limited flexibility in rotation when undergoing radiation therapy. If the patient rotates beyond either one of the tolerance limits, *θ°*(*x*) ± *θ_th_*(*x*), the alarm indicator button *Radiation in Progress* will be activated and an optional audible alarm can be set off. The selection of the tolerance limits for each axis depends on the accuracy of the dosimetric and radiation therapy procedure.

Figure 5 also shows the directional indicators on the control panel, identifying the direction of the patient’s head rotation (e.g., right/left and up/down). This information can be used for adjusting the patient’s head to the reference coordinates.

The GUI-based APOMS control panel offers a number of advanced features that can be enabled. We briefly mention a few of these features in the following paragraphs. We note that these features have not gone under any clinical testing and future studies are required to qualify their efficiency and practicality.

*Record keeping*: Using this feature all changes in orientation can be accurately time stamped and recorded in a database, while the patient is undergoing radiation therapy. Study of such information can potentially allow more precise dosimetric plans for individual patients.

*Automatic reorientation*: By interfacing the APOMS software to external LED panels, it is possible to notify the patient of any excessive head rotation. Furthermore, APOMS can guide the patient to shift to the reference coordinates through a series of audible commands. Clearly, the radiotherapy must be halted while the patient is being redirected to the reference coordinates.

*Radiation interruption*: The APOMS software can easily be interfaced with the linac. Hence, once the patient’s orientation is changed beyond the defined tolerance limits, the system can interrupt the radiation. The linac interrupt circuitry can be activated via wired or wireless communications. Immediate interruption of the radiation can avoid exposure of healthy tissues and unnecessary dose.

*External synchronization*: The APOMS design is capable of supporting additional sensors. APOMS can also receive timing information from external devices. Hence, APOMS can synchronize with external devices and assist skin shift monitoring.

*Independent tolerance limits*: Separate tolerance limits for rotations about each independent axis (e.g., +X or –X) can be enabled in the APOMS software. This can allow more flexible dosimetric planning for patients.

## 6. Experimental Setup and Results

**Figure 6.**
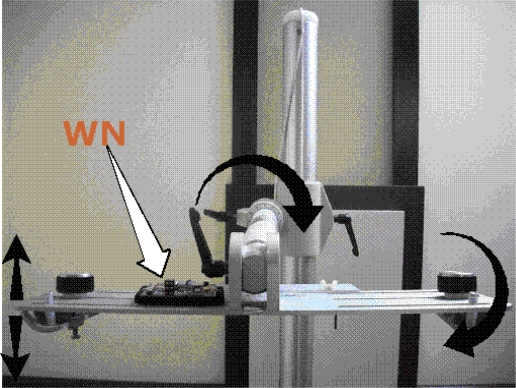
The wireless accelerometer was mounted on the Flexiholder from Huestis Medical.

In order to characterize the accelerometer, we mounted the WN on a Flexiholder [12], as shown in Figure 6. Flexiholder is capable of moving a small platform in three dimensions. Rotation about each individual axis was tested independently. The data sampling rate and the moving average filter were set to 200 Hz and one sample per second, respectively. Each experiment was repeated multiple times to guarantee that the mean results a confidence interval of 15% or better at 90% confidence level. All reported results are referenced to X-, Y-, Z-axis of the accelerometer, as shown in Figure 3(b).

**Figure 7.**
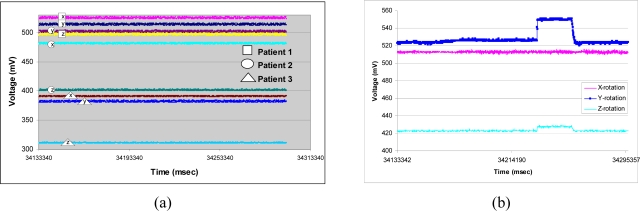
APOMS reading for all three axes: (a) typical voltage readouts for three different patients undergoing radiation for about three minutes; (b) patient tilting her head.

**Figure 8.**
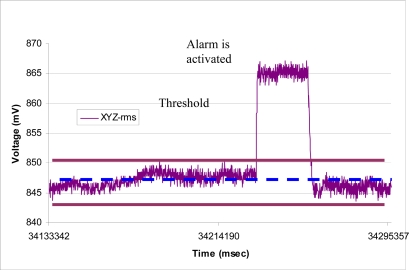
*θ̄*(*xyz*)*_rms_* during a typical treatment session.

Figure 7(a) shows the typical APOMS voltage readouts for three different patients undergoing radiation therapy for about three minutes. This figure indicates that none of the patients changed his/her head orientation during the period of radiation. Figure 7(b) depicts a typical readout of APOMS, while monitoring a patient for a brief period of time after the system is locked. The figure shows that the patient inadvertently tilted her head from the reference (locked) coordinates. Figure 8 shows *θ̄*(*xyz*)*_rms_* for the same scenario. Note prior to the sudden head tilt, initial changes in orientation were within the tolerance limits. When the change in orientation exceeds the threshold (e.g., *θ°* ± *θ_th_*) the alarm is activated.

**Figure 9.**
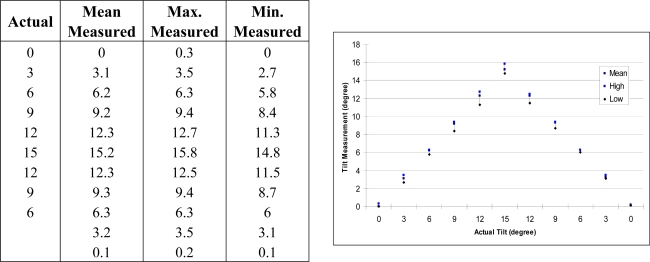
Tabulated results comparing the measured and actual tilt values in degrees for X-axis. Graphical representation of the points is also shown.

The sensitivity and repeatability of APOMS in X-axis are demonstrated in Figure 9. Similar results can be achieved for Y- and Z-axis. For practical purposes, we only tested the changes within 15 degrees. The Min. and Max. values represent the lowest and highest values recorded. These results show that as the tilt angle increases, slightly larger variations from the mean are observed. This is due to the nonlinearity of the accelerometer’s output. Hence, the accelerometer is most sensitive when the sensing axis is closer to zero degrees (zero *g*), and less sensitive when closer to 90 degrees (one *g*).

**Figure 10.**
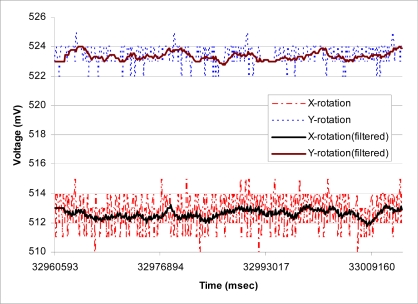
X- and Y-axis readings when the accelerometer is stationary with and without the smoothing filter.

The performance of the moving average filter is shown in Figure 10. In this case, the WN is remained stationary for about one minute and raw data before and after the smoothing filter are recorded. This figure suggests that the X-axis of the accelerometer appears to be noisier than the other two axes. According to our results, the maximum reading variation in X-axis is about 5 mV, corresponding to about half degree, when having a 3.3 V power supply. In general, the noise density increases as the supply voltage drops. Hence, as the system operates for longer period of time, higher noise density is expected. For example, for ADXL330 accelerometer, the noise density at 3.6 V and 2 V are reported to be 230 and 350 $\mu g\sqrt{Hz}$, respectively [8]. Hence, it is important to have WN’s batteries fully charged prior to usage.

**Figure 11.**
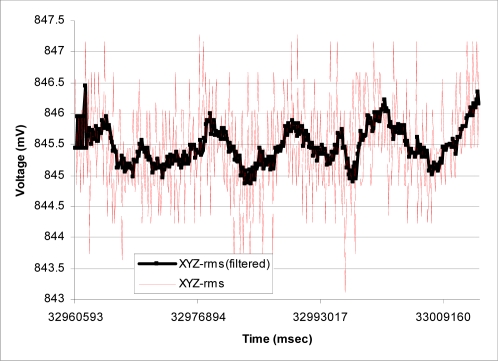
Calculated rms when the accelerometer is stationary with and without the smoothing filter.

Figure 11 compares the rms value of raw and filtered readings for the same scenario. This figure demonstrates that having the moving average filter can significantly reduce the erroneous readings reported by the accelerometer due to noise density and noise voltage in the power supply. Similar results were obtained when we extended the duration of testing to 10 minutes, which is equivalent to a typical radiation therapy session.

In order to fully verify the performance of APOMS, we tested it when the linear accelerator was activated. We examined the impact of the high-energy X-ray source on accelerometers’ readings. For this experiment we recorded the accelerometers’ readings and monitored the received RF signal as the accelerometer was tilted. We recorded the measured tilt angle under three different conditions of linac: no radiation, 6 MV radiations, and 15 MV radiations. In this paper, we use megavoltage (MV) X-Ray, referring to photon beams. In each case, we monitored the amount of the scattered radiation in pC (pico-columbs), detected on the accelerometer and WN’sXbee transceiver using a cavity ionization chamber.

**Figure 12.**
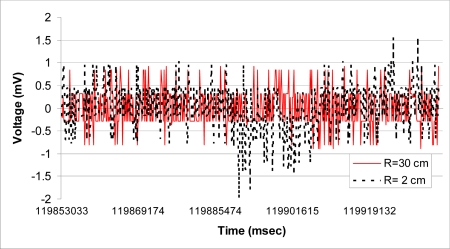
The accelerometer values recorded for R = 30 cm and R = 2 cm.

Figure 12 compares the normalized non-filtered rms readings of the accelerometer for R = 30 cm and R = 2 cm when the accelerometer remain stationary for 1.4 minutes and the linac is generating 15 MV energy. Recall that R represents the distance between location of accelerometer and the collimator generating X-ray radiation, as shown in Figure 2(a).

**Figure 13.**
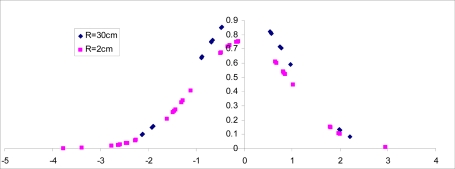
The probability density function (pdf) of a normal population for R = 30 cm and R = 2 cm.

Figure 13 shows the probability density function (pdf) of the accelerometer’s rms reading for similar scenario. Note that the bell curve for R = 2 is slightly shifted and more spread out, suggesting higher variations from the measured mean value. The actual variance for R = 30 cm and R = 2 cm was found to be 0.172 and 0.271, respectively.

We also tested the impact of the high energy X-ray source on the amount of noise generated on Xbee’s RF signal. Our results suggest that the scattered radiation through the collimator radiating 15 MV, does not have any significant impact on WN’s Xbee transceiver performance when it is kept about 30 cm away from the radiation target. Further study may be needed to characterize such distance in more details.

A major issue in APOMS’s performance was found to be the significant attenuation of Xbee’s RF signal through the shielded wall that is designed to block the radiation leakage. The standard shielded wall for CT scanning (also referred to as CAT scanning–Computerized Axial Tomography) with the source generating as high as 15 MV radiation is made of lead-borated-polyethylene and cement with high attenuation factor, depending on the wall’s thickness. Consequently, using Equation (1), for maximum performance, it is necessary to configure the transmission strength of Xbee-PRO for outdoor mode.

## 7. Conclusions

In this paper we have reported the utility of a novel, high-precision, real-time wireless accelerometer-based patient orientation monitoring system (APOMS) in verifying head orientation change of patients undergoing radiation treatment. Important advantageous features of APOMS include ease of use on patients, requiring minimal user training, and needing no change in the typical patient setup.

The design of the APOMS is highly flexible. For example, it can be connected to multiple accelerometers, detecting orientation changes of different parts of the patient’s body, and hence, creating a more complete dataset to correlate the pattern of orientation changes of the internal and external markers. In addition to detecting small, inadvertent orientation change of patient’s head, APOMS can be used in conjunction with different imaging modalities to function in a manner similar to available dynamic tumour tracking systems. For example, the APOMS may be readily used with the fluoroscopy mode of an IGRT-capable linear accelerometer.

Our preliminary clinical test results indicate that the APOMS is highly reliable and can accurately report sub-degree rotational displacements. Furthermore, we showed that under high energy radiation the APOMS performs well, with no degradation to the RF signals between its wireless node and base station.

Although the potential capabilities of the APOMS suggest an improvement over current practice, questions of logistics, durability and clinical functionality still remain. This study was limited to description of the development of the APOMS and an initial evaluation of its feasibility for clinical use. More detailed clinical studies are required to quantify the system’s efficacy and practicality in a simulated environment. Furthermore, we intend to expand the APOMS functionalities using more sophisticated MEMS-based sensors, such as 6-Degrees of Freedom Sensors, in order to develop an inexpensive and flexible head positioning system for patients undergoing multiple radiation treatments.
